# 

**Published:** 1863-04

**Authors:** 


### WORKS PUBLISHED DURING THE QUARTER.

Owing to press of matter, the usual Articles entitled "Medical Gossip" and the "Literary Retrospect" are unavoidably postponed until our next Number.

### 1. *Medicine.*

Bailey.—Letters on the Philosophy of the Human Mind. 3rd Series. By Samuel Bailey. 8vo, cloth, 8s. 6d.

Baines.—Excessive Infant Mortality: How can it be stayed? To which is added, A Short Paper on Infant Alimentation, or Artificial Feeding as a Substitute for Breast-milk, considered in its Physical and Social Aspects. By M. A. Baines. 6d.

Ballard.—On the Convulsive Diseases of Infants. By Thomas Ballard, M.D. 8vo, sewed, 1s.

Brown.—Memories of the Past, and Thoughts on the Present Age. By Joseph Brown, M.D. Post 8vo, cloth.

Chambers.—The Renewal of Life: Clinical Lectures illustrative of a Restorative System of Medicine. By T. K. Chambers, M.D. Second Edition, revised, post 8vo, cloth, 6s. 6d.

Chavasse.—Advice to a Wife on the Management of her own Health, and on the Treatment of some of the Complaints incidental to Pregnancy, Labour, and Suckling. With an Introductory Chapter especially addressed to a Young Wife. By Pye Henry Chavasse, F.R.C.S. Fifth Edition, Fcap. 8vo, 2s. 6d.

Dunn.—Medical Psychology; comprising a Brief Exposition of the leading Phenomena of the Mental States, and of the Nervous Apparatus through which they are manifested. By Robert Dunn, F.R.C.S. Fcap. 8vo, cloth, 3s.

Fleischmann.—Plain and Practical Medical Precepts. By Alfred Fleischmann, M.R.C.S. Second Edition, revised. On sheet, 4d.

Garrod.—The Nature and Treatment of Gout and Rheumatic Gout. By Alfred Baring Garrod, M.D., F.R.S. Second Edition, Enlarged and Revised. Post 8vo, cloth, 15s.

Gordon.—China from a Medical Point of View in 1860 and 1861, to which is added, A Chapter on Nagasaki as a Sanitarium. By Charles A. Gordon, M.D., C.B. With Plans. 8vo, cloth, 10s. 6d.

Harvey.—The Signs and Minor Diseases of Pregnancy. With Remarks on the Use of Chloroform, and the Maternal Management of Infants. By John Harvey, M.D. Fcap. 8vo, cloth, 3s.

Martin.—Special Hospitals. (Reprinted from the "British Medical Journal." By William Martin, F.R.C.S. Post 8vo, 1s.

McClintock.—Clinical Memoirs on Diseases of Women. By Alfred H. McClintock, M.D., F.R.C.S. Illustrated with Wood Engravings from original Drawings. 8vo, cloth, 14s.

Medical Directories (The) for 1863. 8vo, cloth, 10s. 6d.

Medical Register (The) for 1863. Published under the Direction of the General Council of Medical Education and Registration of the United Kingdom, pursuant to "An Act to Regulate the Qualifications of Practitioners in Medicine and Surgery." Royal 8vo, cloth, 4s.

Thompson.—Clinical Lectures on Pulmonary Consumption. By the late Theophilus Thompson, M.D., F.R.S. With Additional Chapters by his Son, E. Symes Thompson, M.D. With Plates, 8vo, cloth, 7s. 6d.

Thudichum.—A Treatise on Gall-Stones: their Chemistry, Pathology, and Treatment. By J. L. W. Thudichum, M.D., M.R.C.P. Coloured Plates, 8vo, cloth, 10s.

Tilt.—A Handbook of Uterine Therapeutics. By E. J. Tilt, M.D., M.R.C.P. Post 8vo, cloth, 6s.

### 2. *Surgery.*

Bryant.—Clinical Surgery. Part IV. On Stricture, Retention of Urine, Stone in the Bladder, and other Diseases of the Urinary Organs. By Thomas Bryant, F.R.C.S. 8vo, 3s. 6d.

Curling.—On the Diseases of the Rectum. By T. B. Curling, F.R.S. Third Edition, Enlarged. 8vo, cloth, 7s. 6d.

Hutchinson.—A Clinical Memoir on certain Diseases of the Eye and Ear, consequent on inherited Syphilis; with an appended Chapter of Commentaries on the Transmission of Syphilis from Parent to Offspring, and its more Remote Consequences. By Jonathan Hutchinson, F.R.C.S. Plates and Woodcuts, 8vo, cloth, 9s.

Lawrence.—Lectures on Surgery. By William Lawrence, F.R.S. 8vo, cloth, 16s.

Lee.—On Syphilis. By Henry Lee, F.R.C.S. Coloured Plates. Second Edition. 8vo, cloth, 10s.

Savage.—The Surgery of the Female Pelvic Organs, in a Series of Plates taken from Nature, with Physiological and Pathological References. By Henry Savage, M.D. London, F.R.C.S. Royal 4to, cloth, 20s.

Wilson.—Diseases of the Skin, a Practical and Theoretical Treatise on the Diagnosis, Pathology, and Treatment of Cutaneous Diseases. By Erasmus Wilson, F.R.S. Fifth Edition, revised, 8vo, cloth, 16s.; with Plates, 34s.

Yearsley.—Deafness Practically Illustrated, being an Exposition of the Nature, Causes, and Treatment of Diseases of the Ear. By James Yearsley, M.D. Sixth Edition, Enlarged, with New Plates. 8vo, cloth, 6s.

### 3. *Anatomy.*

CALLENDER.—Femoral Rupture: Anatomy of the Parts concerned. By George W. Callender. Plates, 8vo, cloth, 4s.

CATALOGUE of the Osteological Portion of Specimens in the Anatomical Museum of the University of Cambridge. 8vo, 2s. 6d.

ELLIS and FORD.—Illustrations of Dissections, in a Series of original Coloured Plates, the size of Life, representing the Dissection of the Human Body. By G. Viner Ellis and G. H. Ford. Folio, Parts I. and II., 3s. 6d. each.

HUXLEY.—Evidence as to Man's Place in Nature. By Thomas Henry Huxley, F.R.S. 8vo, cloth, 6s.

SHEA.—A Manual of Animal Physiology. By John Shea, M.D., B.A. Engravings. Fcap. 8vo, cloth, 5s. 6d.

### 4. *Chemistry.*

BRANDE and TAYLOR.—Chemistry. By William Thomas Brande, D.C.L., F.R.S.L. and E., &c.; and Alfred S. Taylor, M.D., F.R.C.P. London, F.R.S., &c. Fcap. 8vo, cloth, 12s. 6d.

FOWNES.—Fownes' Manual of Chemistry. Edited by H. Bence Jones, M.D., F.R.S.; and A. W. Hofmann, Ph.D., F.R.S. Ninth Edition, Enlarged, with 187 Engravings, 12s. 6d.

HUNTER.—A Report upon some of the Colonial Medicinal Contributions to the International Exhibition, A.D. 1862. By Charles Hunter. 8vo, 2s.

LIEBIG.—The Natural Laws of Husbandry. By Justus von Liebig. Edited by John Blyth, M.D. 8vo, cloth, 10s. 6d.

SAVORY.—Cod-liver Oil: its Purity, Mode of Preparation and Administration. By John Savory, M.S.A. 8vo, 6d.

SUTTON.—A Systematic Handbook of Volumetric Analysis; or, the Quantitative Estimation of Chemical Substances by Measure. Adapted to the requirements of pure Chemical Research, Pathological Chemistry, Pharmacy, Metallurgy, Manufacturing Chemistry, Photography, &c., and for the Valuation of Substances used in Commerce, Agriculture, and the Arts. By Francis Sutton, F.C.S. Engravings. Post 8vo, cloth, 7s. 6d.

